# Kinetic Factors Related to the Side Hop Test Time in Healthy Individuals

**DOI:** 10.1155/tsm2/3241325

**Published:** 2025-09-26

**Authors:** Kyoya Ono, Shojiro Nozu, Takuya Yoshida, Masamichi Okudaira, Kazuki Ota, Satoru Tanigawa

**Affiliations:** ^1^JUBILO CO., LTD., Shizuoka, Japan; ^2^Faculty of Health and Sports Science, Juntendo University, Chiba, Japan; ^3^Department of Sports Sciences, Japan Institute of Sport Sciences, Tokyo, Japan; ^4^Faculty of Education, Iwate University, Iwate, Japan; ^5^Faculty of Sport Science, Yamanashi Gakuin University, Yamanashi, Japan; ^6^Faculty of Health and Sports Sciences, University of Tsukuba, Ibaraki, Japan

**Keywords:** ankle, cutting movements, functional performance test, kinetics, return to sports

## Abstract

The side hop test (SHT) has been proven to be a highly valid assessment tool for the ankle joint. However, previous studies have not revealed which joints affect the SHT time. Thus, interpreting the SHT time correctly may be challenging. Investigating factors that determine the SHT time would provide important basic information regarding the SHT. Therefore, we aimed to investigate the kinetic factors related to the SHT time in healthy individuals. Twenty healthy male college soccer players formed our study population. Three-dimensional motion analysis was conducted during the SHT. The SHT time and peak torque power (hip, knee, and ankle) during the medial hop (MC) and lateral hop contact (LC) phases in the SHT were calculated. The relationship between the SHT time and peak torque power was examined using Pearson's correlation coefficient. In terms of concentric power, significant negative correlations were found between the SHT time and peak concentric hip power in the sagittal (*r* = −0.77 to −0.67) and frontal planes (*r* = −0.74 to −0.47) in each phase. For eccentric power, significant positive correlations were found between the SHT time and peak eccentric ankle power in the sagittal plane (*r* = 0.48–0.50) in each phase. In addition, significant positive correlations were found between the SHT time and peak eccentric hip power in the frontal plane (*r* = 0.55) in MC, peak eccentric hip power in the sagittal plane (*r* = 0.63), and eccentric ankle power in the frontal plane (*r* = 0.52) in LC. Our results showed that concentric and eccentric power were important for the hip and ankle joints, respectively, and different functional requirements depended on the joint. Based on these findings, clinicians can use the SHT to assess an athlete's condition and appropriately determine their safe return to sports.

## 1. Introduction

Clinicians use simple performance tests to assess the rehabilitation progress and conditions of athletes. These tests are commonly known as functional performance tests (FPTs) and comprise movements that exert load on the lower extremity, such as hopping, jumping, or cutting. FPTs can be used to assess the components of sports performance (strength, power, and agility) and determine the time to return to sports (RTS) by providing quantitative and qualitative information regarding athletic movements [1–4]. The advantage of FPTs is that they can be conducted quickly with minimal personnel and equipment requirements [2]. Quantitative results obtained from FPTs are insufficient to predict the occurrence of sports injuries. However, they may indicate the causal relationships with injury risk [5]. Therefore, assessing the athletes' conditions using FPTs is important in preventing injury or determining safe RTS for patients.

The side hop test (SHT) is a representative FPT with multiple cutting movements. In this test, a participant is instructed to hop as fast as possible with a single leg on two parallel lines which are 30 cm wide, and the time to complete 10 round trips is recorded [6]. The SHT has been reported to be capable of differentiating between those with and without chronic ankle instability (CAI) [7], as the SHT time is delayed in individuals with CAI [6, 8–10]. The kinematics and muscle activity during the SHT have been reportedly investigated in a laboratory to better understand the SHT [11–13]. However, previous studies have mainly focused on the ankle joint. The findings of movement characteristics of the hip and knee joints are limited. In addition, there is a paucity of information regarding which joints in the lower extremity contribute to the SHT time. Previous studies on single-leg cutting have reported that ankle plantar flexion moment, ankle joint power, hip extension moment, and hip joint power are related to a faster cutting performance [14–16]. Based on these findings, it can be considered that the SHT may also require contributions from each joint. Taking the kinetic chain of the lower extremity into consideration, findings limited to the ankle joint are considered insufficient for a comprehensive understanding of the SHT. Correct interpretation of the results without basic information regarding which joint affects the results obtained from the SHT may be a challenging and arduous task. Therefore, investigating the contribution of each joint to the SHT time by focusing on all the lower extremity joints is important for effectively using the SHT.

Centering on the kinetics of the lower extremity joints would be useful in investigating the factors that determine the SHT time. Since the SHT involves repetitive landings and lateral push-offs with a single leg, effective impact attenuation and propulsion at the lower extremity joints are critical to the performance of the SHT [17]. Previous studies have reported that the ankle plantar flexors, knee, and hip extensor muscles contribute to impact attenuation after ground contact by contracting eccentrically [18]. There is a possibility that significant eccentric contractions in the lower extremity muscle groups could be observed in the SHT. However, whether these factors relate to faster SHT times remains unknown at present. Therefore, it is necessary to focus on torque power, which could reflect concentric and eccentric contractions, to clarify the specific role of the lower extremity joints in the SHT. Investigating the relationship between the SHT time and the torque power of the lower extremity joints could help in the identification of factors that clearly explain the SHT time and provide valuable insight into the SHT.

Since chronic disorders, such as CAI, could alter the movement characteristics of the lower extremities, it is required to focus on individuals without CAI to provide primary findings related to the SHT. Therefore, this study aimed to investigate the kinetic factors related to the SHT time in healthy individuals, focusing on the entire lower extremity. Considering the fact that previous studies reported the contribution of the hip joint to cutting movements with a single leg [14–16] and that the SHT includes cutting movements, we hypothesized that the torque power of the hip and ankle joints may equally relate to the SHT time. This study is an expanded and updated version of the proceedings from the 39^th^ International Society of Biomechanics in Sport Conference [19]. The proceeding simply investigated the correlation between the SHT time and peak torque in the lower extremity. In contrast, this study mainly investigated the correlation between the SHT time and peak torque power in the lower extremity and also presented time series data on kinematics and kinetics during the ground contact phase of the SHT, providing insightful information.

## 2. Materials and Methods

### 2.1. Ethical Considerations

All the procedures performed in this study were conducted in accordance with the tenets of the Declaration of Helsinki. This study was approved by the University of Tsukuba Research Ethics Committee (Ref. No. 021-122). The purpose of the study was explained to all the participants, and their consent was obtained prior to the commencement of the study.

### 2.2. Participants

This study included 20 college soccer players (mean ± SD; age = 20.25 ± 1.41 years, height = 172.67 ± 4.93 cm, weight = 67.72 ± 5.41 kg). Participants were excluded from the study if they had any one of the following: previous musculoskeletal surgery of the lower extremity, previous lower extremity fracture requiring repair, and musculoskeletal injury of the lower extremity requiring at least one day of physical inactivity within the past 3 months before the measurement.

### 2.3. Procedures

Participants performed the SHT in an indoor laboratory. The participants were instructed to hop as fast as possible with a single leg on two parallel lines which were 30 cm wide, and the time to complete 10 round trips was recorded [6]. The test leg was defined as the leg the participants used to kick the ball while playing soccer. All the participants performed the SHT with their right leg. Participants were also instructed to wear familiar indoor shoes for the experiment. Participants commenced the test by standing on a single leg, putting both hands on their hips, and hopping laterally at the start signal. They then repeated the lateral hopping with maximum effort and completed a 10-round trip. We modified the method given by Yoshida et al. [12, 13] and considered that if the supporting leg stepped on the line more than twice, the free leg touched the ground, or the hands left the hips, the trial was a failure. After a general warm-up, participants were instructed on how to perform the trial before data collection, and they practiced accordingly. The SHT was conducted until three successful trials were recorded, and the fastest performance trial was used for the analysis. A rest period of at least 1 min was provided between trials to minimize the effects of fatigue.

Following the method of Suzuki et al. [20], 47 retroreflective markers (14 mm diameter) were attached to the anatomical landmarks on the participant's entire body. Three-dimensional (3D) coordinates data were collected at 250 Hz using 10 Vicon MX + cameras (Vicon Motion System, Ltd., Oxford, UK). Two force platforms (9287C, Kistler Instrumente AG, Winterthur, Switzerland) were used to collect ground reaction force (GRF) data at 1000 Hz. The global coordinate system defined the *x*-axis (anterior/posterior) in front of the participants at the beginning of the trial, the *y*-axis (medial/lateral) as perpendicular to the *x*-axis, and the *z*-axis (upward/downward) for the vertical direction. The SHT time and ground contact time (GCT) were calculated using GRF data. Ground contact was defined as when the vertical component of GRF exceeded 20 N, and take-off was defined as when the vertical component of GRF fell below 20 N.

### 2.4. Data Analysis

The obtained 3D coordinates for each body part and GRF data were temporally synchronized using Vicon Nexus software (Vicon Motion System, Ltd., Oxford, UK). The 3D coordinates and GRF data were smoothed at 10 Hz using a fourth-order Butterworth low-pass digital filter. GRF data were interpolated at 250 Hz using a spline function to synchronize with the 3D coordinate data. Rigid-body link models (foot, shank, thigh, and pelvis segments) were created from the 3D coordinates of the markers, in accordance with the previously described methods [21, 22]. The joint angular velocity was estimated by time differentiation of the joint angles, which was calculated using the Cardan rotation sequence. Joint torques were calculated as net internal torques of the hip, knee, and ankle joints by inverse dynamic analysis. Torque power in the sagittal and frontal planes was calculated by multiplying joint angular velocity and torque. Joint torque and torque power were normalized according to each participant's body weight.

In accordance with the definition given by Yoshida et al. [12, 13], GRF data were used to divide the SHT into two ground phases: the medial hop contact (MC) and lateral hop contact (LC) phases. MC was the medial phase, and LC was the lateral phase relative to the test leg. All variables in this study were calculated as the average of eight of the 10-round trips in the SHT, excluding the 1^st^ and 10th-round trips [12, 13]. All variables were also calculated for MC and LC data. In order to present time series average data for joint angular velocity, torque, and torque power, data were normalized from the ground contact (0%) to take-off (100%) as 100%. Data analysis was performed using MATLAB R2021b (MathWorks Inc., Natick, MA, USA).

### 2.5. Statistical Analysis

Pearson's correlation analysis was used to investigate the relationships between the SHT time, GCT, and peak torque power in the sagittal and frontal planes during the SHT. An *r* value ranging between 0 and 0.3 (−0.3) was interpreted as small, 0.31 (−0.31)–0.49 (−0.49) as moderate, 0.50 (−0.50)–0.69 (−0.69) as large, 0.70 (−0.7)–0.89 (−0.89) as very large, and 0.90 (−0.90)–1.00 (−1.00) as near perfect [23]. Statistical processing was performed using IBM SPSS Statistics (Version 27 package; IBM). Statistical significance was set at *p* < 0.05.

## 3. Results

**Figure 1:**
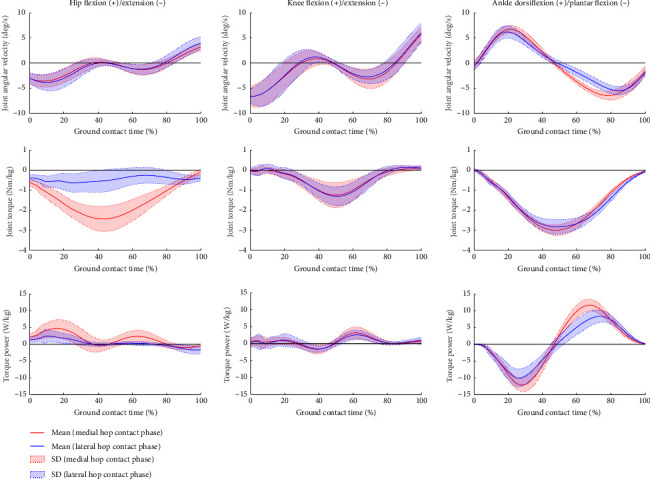
Time series data of joint angular velocity, joint torque, and torque power in the sagittal plane during the side hop test.

**Figure 2:**
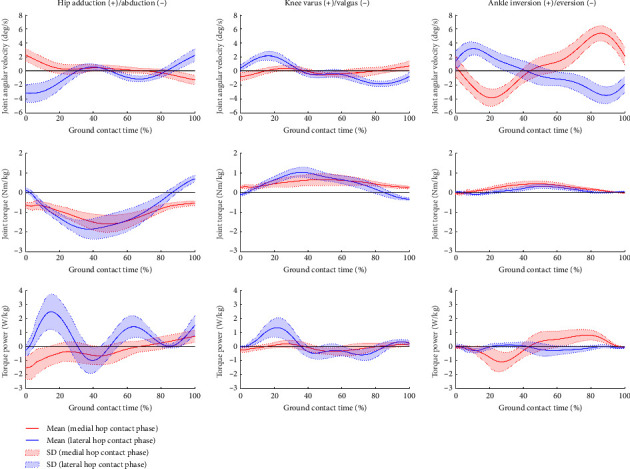
Time series data of joint angular velocity, joint torque, and torque power in the frontal plane during the side hop test.

The average time to perform the SHT was 6.48 ± 0.58 (range: 5.27–7.24) s. Descriptive statistics for GCT and peak torque power during the SHT are shown in Table 1, and the correlations between the SHT time and each variable are depicted in Table 2. The time series average data of the joint angular velocity, torque, and torque power in MC and LC are shown in Figures 1 and 2.

Significant positive correlations were found between the SHT time and GCT in MC and LC (*r* = 0.70 and *r* = 0.72, respectively; *p* < 0.01) (Table 2). In terms of the relationship between the SHT time and peak torque power, a significant negative correlation was found between the SHT time and peak concentric hip power in the sagittal (*r* = −0.77, *p* < 0.01) and frontal planes (*r* = −0.47, *p*=0.04) in MC (Table 2). In addition, significant positive correlations were found between the SHT time and peak eccentric ankle power in the sagittal plane (*r* = 0.50, *p*=0.03) and eccentric hip power in the frontal plane (*r* = 0.55, *p*=0.01) in MC (Table 2).

In LC, significant negative correlations were found between the SHT time and peak concentric hip power in the sagittal (*r* = −0.67, *p* < 0.01) and frontal planes (*r* = −0.74, *p* < 0.01) (Table 2). In addition, significant positive correlations were found between the SHT time and peak eccentric ankle power in the sagittal (*r* = 0.48, *p*=0.03) and frontal planes (*r* = 0.52, *p*=0.02) and eccentric hip power in the sagittal plane (*r* = 0.63, *p* < 0.01) in LC (Table 2).

## 4. Discussion

The results of this study confirmed the fact that the SHT time correlates significantly with hip and ankle torque power, indicating that it is important for the hip joint to function concentrically and the ankle joint to function eccentrically. To the best of our knowledge, this study is the first to investigate kinetic parameters related to the SHT time, focusing on the entire lower extremity. Although previous studies have shown that individuals with CAI exhibit different power exertion patterns during cutting compared to healthy individuals [21, 24], it was impossible to interpret whether these differences affected the SHT time. Therefore, to interpret the SHT time correctly, it was necessary to examine the kinetic factors related to the SHT time in healthy individuals, eliminating the effects of CAI. The findings of this study provide foundational information for interpreting the SHT time for the appropriate assessment of athletes' conditions and determination of RTS.

This study showed that concentric hip power is important in the sagittal and frontal planes. Previous studies have reported that faster cutting movements require resistance to hip flexion just after ground contact [15]. Although impact attenuation by hip flexion motion seems more efficient compared to other joints considering the skeletal muscle structure, it could prolong the GCT during ground contact. Moreover, previous studies have reported a relationship between GCT during the cutting task and performance [14, 25, 26]. This was consistent with the results of our study (Table 2). Based on these findings, it can be considered that concentric hip power in the sagittal plane may prevent hip flexion movements that could result in prolonged GCT after ground contact. In addition, the hip adductor/abductor muscles play an important role in controlling the body position and balance during the cutting task [20]. Furthermore, a smaller pelvic depression on the swinging leg has been related to greater cutting performance [14]. In the present study, hip abduction torque was observed during most of the ground contact in each phase (Figure 2), suggesting that concentric hip power in the frontal plane maintains the pelvic position and also enhances the stability of the support leg. Thus, concentric hip power in each plane contributes to shorter GCT and postural control in the SHT, leading to faster SHT time.

In contrast to the hip joint, eccentric ankle power, particularly in the sagittal plane, was found to be more important for faster SHT time. However, Marshall et al. reported a significant correlation between cutting performance and concentric ankle power [14], which was not in concordance with the results of the present study. In contrast to the typical cutting task, the SHT distance was set at 30 cm and required multiple round trips. It is possible that hopping excessively beyond the set distance in the SHT results in a delayed time. Thus, eccentric ankle power may be more important for effective and fast impact attenuation in the SHT than concentric ankle power. Furthermore, it was found that the eccentric hip power in the sagittal plane could contribute to hip control immediately before take-off in LC (Figure 1). Several studies have reported that hip extension movement is important for high performance in single-leg lateral jump and sidestep [27–29]. However, since excessive propulsive force could interfere with the efficiency of SHT, eccentric hip power in the sagittal plane in LC could contribute to the maintenance of hip flexion for efficient movement into MC. Thus, eccentric ankle power in the sagittal plane may contribute to impact attenuation in MC and LC phases. Moreover, eccentric hip power in the sagittal plane supports the efficiency of SHT in LC.

The contribution to impact attenuation in the frontal plane unexpectedly exhibited a phase-specific pattern. The eccentric ankle power in the frontal plane could contribute to impact attenuation in LC. A previous study reported that ankle eversion motion attenuated impact for appropriately controlling the center of mass in MC [12]. However, the study did not investigate whether the ankle eversion pattern in MC leads to faster SHT. Thus, it was not considered to be related to the SHT time based on the present study. Since ankle sprains frequently occur during side cutting [30], this injury pattern would reflect the importance of impact attenuation by the ankle joint in LC. Conversely, the eccentric hip power in the frontal plane could contribute to impact attenuation in MC. Suzuki et al. reported that internal hip abduction torque prevents hip displacement since external hip adduction torque occurs during crossover cutting [20]. Although this study did not investigate the similarity between the movement of MC and the crossover step, as the SHT requires control of the lower extremities in the frontal plane, the eccentric hip power in the frontal plane would contribute to impact attenuation in MC. Thus, impact attenuation would primarily be performed by the eccentric ankle power in the sagittal plane (Figure 1). However, the eccentric power in the frontal plane would partially function in coordination, and specific movement patterns would be observed depending on the phase.

This study had a few limitations, and thus, the results should be interpreted cautiously. First, only soccer players who regularly engage in high-intensity sports activities were included in this study. Since soccer involves frequent cutting movements in all directions, the participants were assumed to be good at lateral cutting movements. Second, compared to the previous study [12], the participants recorded faster SHT times (6.48 ± 0.58 vs. 7.29 ± 0.58). Since this study's results do not indicate the kinetic characteristics of the SHT in athletes at all levels, it is necessary to investigate the biomechanical characteristics of the SHT in various athletes participating in other sports activities in the future.

## 5. Conclusion

This study indicated that the torque power of the ankle and hip joints was related to the SHT time. We found that concentric power and eccentric power were important in the hip and ankle joints, respectively, and different functional requirements were dependent on the joint during the SHT. This study revealed kinetic factors that could affect the SHT time. Furthermore, the study also provided insight that emphasized the importance of considering the entire lower extremity, not just a single joint. Thus, clinicians can use the SHT to assess an athlete's condition and appropriately determine safe RTS based on the findings of this study.

## Figures and Tables

**Table 1:** Descriptive statistics for ground contact time and peak torque power during the side hop test.

Variables	MC		LC	
	Mean ± SD	Range	Mean ± SD	Range
Ground contact time (ms)	191.50 ± 16.46	162.00–224.00	198.35 ± 16.70	171.00–226.00
Concentric torque power (W/kg)				
Sagittal plane				
Hip	5.67 ± 2.64	2.37–10.54	3.01 ± 2.23	0.64–10.20
Knee	4.31 ± 1.39	2.15–7.97	3.95 ± 1.57	1.77–9.63
Ankle	12.06 ± 1.69	8.92–15.58	8.66 ± 1.91	5.61–14.23
Frontal plane				
Hip	1.17 ± 0.40	0.57–1.96	3.06 ± 0.98	1.30–4.62
Knee	0.47 ± 0.23	0.16–1.05	1.47 ± 0.63	0.76–2.89
Ankle	1.19 ± 0.58	0.40–2.19	0.47 ± 0.21	0.19–0.95
Eccentric torque power (W/kg)				
Sagittal plane				
Hip	−2.11 ± 0.93	−4.03–−0.68	−2.01 ± 1.18	−5.73–−0.58
Knee	−2.63 ± 1.58	−7.55–−0.76	−2.64 ± 1.09	−6.20–−0.83
Ankle	−12.80 ± 1.94	−15.81–−8.78	−10.72 ± 2.73	−18.77–−6.48
Frontal plane				
Hip	−2.08 ± 0.80	−4.07–−0.58	−1.42 ± 0.69	−2.74–−0.29
Knee	−0.67 ± 0.25	−1.11–−0.26	−1.04 ± 0.26	−1.68–−0.55
Ankle	−1.38 ± 0.79	−3.71–−0.50	−0.77 ± 0.34	−1.76–−0.30

*Note:* MC = medial hop contact phase; LC = lateral hop contact phase.

**Table 2:** Correlations between the side hop test time and each variable.

Variables	MC		LC	
	*r*	*p*	*r*	*p*
Ground contact time	0.70^†^	< 0.01	0.72^†^	< 0.01
Concentric torque power				
Sagittal plane				
Hip	−0.77^†^	< 0.01	−0.67^†^	< 0.01
Knee	−0.04	0.88	−0.37	0.11
Ankle	−0.21	0.38	−0.20	0.39
Frontal plane				
Hip	−0.47^∗^	0.04	−0.74^†^	< 0.01
Knee	−0.21	0.38	−0.01	0.97
Ankle	−0.23	0.34	−0.42	0.06
Eccentric torque power				
Sagittal plane				
Hip	0.36	0.12	0.63^†^	< 0.01
Knee	0.04	0.87	0.22	0.34
Ankle	0.50^∗^	0.03	0.48^∗^	0.03
Frontal plane				
Hip	0.55^∗^	0.01	−0.14	0.55
Knee	0.09	0.72	0.31	0.18
Ankle	0.25	0.30	0.52^∗^	0.02

*Note:* MC = medial hop contact phase; LC = lateral hop contact phase.

^∗^ *p* < 0.05.

^†^ *p* < 0.01.

## Data Availability

The data that support the findings of this study are available on request from the corresponding author. The data are not publicly available due to privacy or ethical restrictions.
